# Pattern of nucleotide variants of *TP53* and their correlation with the expression of p53 and its downstream proteins in a Sri Lankan cohort of breast and colorectal cancer patients

**DOI:** 10.1186/s12885-020-6573-5

**Published:** 2020-01-30

**Authors:** Vahinipriya Manoharan, Eric Hamilton Karunanayake, Kamani Hemamala Tennekoon, Sumadee De Silva, Ahamed Ilyas Ahamed Imthikab, Kanishka De Silva, Preethika Angunawela, Sameera Vishwakula, John Lunec

**Affiliations:** 1 Institute of Biochemistry Molecular Biology and Biotechnology, University of Colombo, 90, Cumaratunga Munidasa Mawatha, Colombo 3, Sri Lanka; 2 National Cancer Institute, Maharagama, Sri Lanka; 3 Department of Pathology, Faculty of Medicine, University of Colombo, 25 Kynsey Road, Colombo 8, Sri Lanka; 4 Department of Statistics, Faculty of Science, University of Colombo, Colombo 3, Sri Lanka; 5 Northern Institute for Cancer Research, Newcastle University, Paul O’Gorman Building, Framlington Place, Newcastle upon Tyne, NE2 4AD UK

**Keywords:** Breast cancer, Colorectal cancer, Sri Lanka, *TP53*, Genetic variation, Immunohistochemistry

## Abstract

**Background:**

Breast cancer (BC) is known to be the most common malignancy in females whereas colorectal cancer (CRC) incidence also higher in both genders in Sri Lanka. *TP53* is an important tumour suppressor gene and its somatic mutations are reported in approximately 27% of BC and 43% of CRC cases. Analysis of *TP53* gene variants not only provides clues for the aetiology of the tumour formation, but also has an impact on treatment efficacy. The current study was conducted to investigate the pattern of *TP53* variants in patients with BC and CRC from Sri Lanka.

**Methods:**

30 patients with BC, 21 patients with CRC and an equal number of healthy controls were screened for mutational status of *TP53* by polymerase chain reaction (PCR) followed by direct sequencing. In addition, a subset of these samples were analysed for the protein expression of p53 and comparison made with the mutational status of *TP53*. We also analysed the protein expression of p21 and MDM2 as potential indicators of p53 functional status and compared it with the protein expression of p53. Additionally, hotspot codons of the *KRAS, BRAF* and *PIK3CA* genes were also analysed in a subset of CRC patients.

**Results:**

Twenty seven sequence variants, including several novel variants in the *TP53* gene were found. Nine BC and seven CRC tumour samples carried pathogenic *TP53* variants. Pathogenic point missense variants were associated with strong and diffuse positive staining for p53 by immunohistochemistry (IHC), whereas, wild type *TP53* showed complete absence of positive IHC staining or rare positive cells, regardless of the type of cancer. There was no direct correlation between p21 or MDM2 expression and p53 expression in either BCs or CRCs. Four of the CRC patients had pathogenic hotspot variants in *KRAS*; three of them were on codon 12 and one was on codon 61.

**Conclusion:**

The prevalence of pathogenic somatic *TP53* variants was 31 and 33.33% in the studied BC and CRC cohorts respectively. All of them were located in exons 5–8 and the pathogenic missense variants were associated with strong immuno-positive staining for p53.

## Background

BC and CRC are commonly diagnosed malignancies worldwide [1, 2]. According to cancer incidence data, 2014 by the National Cancer Control Programme, BC is the leading cancer among Sri Lankan women accounting for 25.2% of total cancers and CRC ranks fourth (6.9%) and fifth (7.3%) in males and females respectively [3]. Early menarche, late menopause, being nulliparous, lack of breast feeding, use of oral contraceptives, and a family history of BC or other cancers are some of the major risk factors associated with BC [4] while risk factors for CRCs include age, obesity, dietary factors, smoking, alcoholism and personal history of adenomatous polyps/inflammatory bowel diseases [5].

*TP53*, a tumour suppressor gene is one of the key factors involved in tumour development, progression and prognosis. Somatic mutations in the *TP53* gene are reported in approximately 26.51% of BC and 43.32% of CRC cases [1, 6]. The *TP53* gene contains 11 exons and 10 introns and is located on the short arm of chromosome 17. The p53 protein is a phosphoprotein of 393 amino acid (55 kDa) which includes an amino-terminal acidic transcription activation domain (1–67), a proline rich region (67–98), a core DNA binding domain (98–303), a nuclear localization signal-containing region (303–323), an oligomerisation domain (323–363) and a C-terminal basic domain (363–393). It acts as a transcription factor for several target genes. The *CDKN1A* gene, major transcriptional target of p53, codes for the p21 cyclin-dependent kinase inhibitory protein which causes cell cycle arrest. *MDM2* is another important transcriptional target gene of p53, where the MDM2 protein controls the level of p53 by a negative auto–regulatory feedback loop in which MDM2 binds to and ubiquitinates p53, targeting it for proteasomal degradation [7].

According to the COSMIC database, more than 50% of the *TP53* alterations are missense mutations. This is followed by non-sense mutations contributing to about 10% of total *TP53* alterations [6]. The functional status of p53 has an impact on treatment efficacy [8]. Thus, recognition of the functional status of p53 may benefit in the selection of treatment option and prognostication of treatment efficacy. Identification of hotspot regions of *TP53* variants is useful to prioritize screening of such regions prior to treatment in a resource limited setting such as Sri Lanka.

Most research on p53 including trends of incidence, genetic analysis and treatment response have been carried out in developed countries, while analysis of such trends and patterns in developing countries including Sri Lanka are limited. Since the types of *TP53* alterations and their frequencies have been suggested to be influenced by geographical factors and ethnicity, the current study intended to establish the mutation spectrum of *TP53* in Sri Lankan BC and CRC patients [9, 10]. This is the first report on *TP53* alterations in Sri Lankan patients with sporadic BC and CRC.

In addition, association of BCs with CRCs is controversial, as some studies have suggested that BC survivors are at a higher risk of developing CRC due to risk factors such as obesity and the level of exogenous and endogenous sex hormones [11, 12] while other studies have proposed that there are no such associations [13]. In this study, we compared the mutation spectrum of *TP53* among BCs and CRCs to evaluate their genetic basis.

Furthermore, immunohistochemistry (IHC) was carried out to measure the protein expression of p53 and to correlate the immuno-detection of p53 with the mutational status of *TP53* gene. We also studied the expression of p53 downstream targets and compared those with the immune-detection of p53.

## Methods

### Recruitment of the participants for the study and processing of the samples

Ethical clearance (EC/14/160) was granted by the Ethics Review Committee of the Faculty of Medicine, University of Colombo, Sri Lanka. A total of 92 participants were recruited, which includes 30 patients with BC, 21 patients with CRC and 41 healthy controls (30 females, 11 males) without any personal or family history of cancer.

Prior to the recruitment, the study was explained and written informed consent was obtained from the patients and healthy controls. Clinical and socio- demographic data of the participants were collected via medical reports and questionnaires respectively.

One part of the surgically excised tumor tissues was placed in 10% formalin to make Formalin Fixed Paraffin Embedded (FFPE) blocks, while the other part was placed immediately in Allprotect® Tissue Reagent (QIAGEN, cat no. 76405, Hilden, Germany) and stored at-20 °C until processed.

Genomic DNA extraction from the excised tumour piece of patients and from blood of healthy controls was carried out as in Manoharan et al., 2019 [14]. PCR amplification of *TP53* exon and flanking genomic DNA sequences was performed. The nucleotide sequence of PCR primers used and reaction conditions have been previously reported [14]. Wizard® SV Gel and PCR Clean-Up kit (Promega) was used to purify the PCR products. Direct sequencing was carried out for purified products using the BigDye® Terminator v3.1 kit (Thermo Fisher Scientific, Waltham, MA USA) and an Applied Biosystems™ 3500Dx Genetic Analyzer (ThermoFisher Scientific).

The sequencing results were analyzed through BioEdit® software by aligning with the Human NCBI *TP53* reference sequence (Genbank accession number - NC_000017) and confirmed further using Mutation Surveyor®V4.0.9 and Alamut® Visual 2.7.2 Documentation. Guidelines of the Human Genome Variation Society (HGVS) nomenclature (http:/www.hgvs.org/mutnomen/) were used to name the identified sequence variants.

### Analysis of sequence variants

The following databases were used to check the identified sequence variants for previous reporting: Catalogue Of Somatic Mutations in Cancer (COSMIC) (http://cancer.sanger.ac.uk/cosmic); NCBI (https://www.ncbi.nlm.nih.gov/); IARC TP53 (http://p53.iarc.fr/); Ensembl (https://asia.ensembl.org/index.html); the p53 website (https://p53.fr/tp53-database).

Pathogenicity of exonic variants was analysed using comparative programs of missense prediction; Align GVGD (http://agvgd.hci.utah.edu/agvgd_input.php); SIFT (http://sift.jcvi.org/www/SIFT_seq_submit2.html); MutationTaster (http://www.mutationtaster.org/); PolyPhen-2 (http://genetics.bwh.harvard.edu/pph2/); Provean (http://provean.jcvi.org/seq_submit.php). Gene splicing was assessed using Human Splicing Finder V3.0 (http://www.umd.be/HSF3/) and splicing window of Alamut® Visual software. Data on structural and functional activity of the p53 available on IARC TP53 database such as transcriptional activity and Dominant Negative Effect was also considered for pathogenicity determination of the exonic variants identified [15]. Classification of all variants was done according to the standards and guidelines of American College of Medical Genetics [16].

Structure based activity prediction for the novel exonic variants was done using protein structure comparison software TM align (https://zhanglab.ccmb.med.umich.edu/TM-align/). The X-ray diffraction structure of wild type p53 protein complexed with DNA (PDB ID – 1TUP) was used to identify the structural position of variant sequences. The structure of the protein with novel variants was built using SWISS-MODEL (https://swissmodel.expasy.org/).

### Immuno expression of p53, p21 and MDM2

IHC characterization was performed on representative FFPE tumour sections of thirteen BC and fourteen CRC cases randomly selected, to evaluate the immuno expression of p53, p21, and MDM2. The commercially available primary antibodies used were mouse monoclonal Anti-Human p53 clone DO-7 (Agilent DaKo, Santa Clara, USA), Rabbit monoclonal p21 Waf1/Cip1 2947S (Cell signalling technology, Danvers, MA, USA) and mouse monoclonal Anti-MDM2 OP46 (MerkMillipore, Massachusetts, USA) for detecting of p53, p21 and MDM2 respectively. Trials were performed to optimise the concentration, incubation time of the primary antibodies and antigen retrieval buffer (Citrate, pH = 6 and Tris, pH = 9). The best outcomes were used for the samples and the optimised conditions are tabulated in Additional file 1: Table S1. The paraffin-embedded pellets of MDM2 inhibitor treated (5 μM Nutlin-3) MCF7 cells was used as a positive control for p53 and p21 antibodies and paraffin-embedded pellets of MDM2 inhibitor treated (10 μM Nutlin-3) SJSA cells was used as a positive control for the MDM2 antibody. The general IHC conditions have been previously reported [14]. AperioScanScope® CS System (Aperio Technologies, Bristol, UK) and Spectrum™ image management software were used to visualize the images of the IHC stained slides and the images were finally analysed as described in Manoharan et al., 2019 [14].

### Analysis of hotspot regions of KRAS, BRAF and PIK3CA

PCR specific primers were designed to amplify the hotspot regions of *KRAS* (Codon 12, 13, 61 and 146), *BRAF* (codon 600) and *PIK3CA* (codon 1047). 17 CRC samples were subjected to PCR amplification followed by direct sequencing. The results obtained were analysed in the same manner as for *TP53.*

### Statistical analysis

The Pearson’s chi-squared test and Spearman’s Rank Correlation tests were done to find the strength and the direction of the associations. A *p*-value < 0.05 was considered as statistically significant at 5% level.

## Results

### Baseline characteristics of the study participants

Mean (±SD) age of the studied patient cohort was 59.24 (±10.16) years for BC and 60.29 (±11.45) for CRC, 48.9 (±13.91) for healthy female controls and 49.01 (±17.14) for healthy male controls. The baseline characteristics of the BC and CRC patients are summarized in Tables 1 and 2 respectively. The majority of both BC and CRC patient population represents the Sinhalese ethnicity and were over 40 years in age.

**Table 1:** Clinicopathological characteristics of breast cancer patients and healthy controls

Characteristics	Total Number of patients	Number of patients with Wild type *TP53*	Number of patients with mutated *TP53*	Number of healthy controls
Ethnicity				
Sinhalese	25	18	7	24
Tamil	1	1	0	5
Muslims	2	2	0	0
Burgher	2	0	2	1
Age at study entry				
< 40 years	1	1	0	9
40–60 years	14	11	3	13
> 61 years	15	10	5	8
Body Mass Index				
Underweight	2	2	0	2
Ideal	9	7	2	9
Overweight	5	4	1	4
Pre obese	10	6	4	11
Obese	4	2	2	4
Tumour type				
Invasive Ductal Carcinoma	29	20	9	N/A
Invasive Lobular Carcinoma	1	1	0	N/A
Breast affected				
Left	11	7	4	N/A
Right	17	13	4	N/A
both	2	1	1	N/A
Menstrual status				
Pre menopausal	7	5	2	19
Post menopausal	23	16	7	11
Pregnancy history				
Nulliparous	7	4	3	9
1–3 children	21	16	5	20
> 3 children	2	1	1	1
Breast Feeding history (Total)				
No breast feeding	7	4	3	9
< = one year	4	3	1	8
> one year	18	13	5	13
History of cancer				
Previous history	6	3	3	N/A
Family history	11	8	3	N/A
Codon 72 polymorphism				
Arginine	12	10	2	12
Proline	7	2	5	5
Arginine/ Proline	11	9	2	13

**Table 2:** Baseline characteristics of colorectal cancer patients and healthy controls

Characteristics	Total Number of patients	Number of patients with Wild type *TP53*	Number of patients with mutated *TP53*	Number of healthy controls
Sex				
Male	14	12	2	11
Female	7	2	5	10
Ethnicity				
Sinhalese	19	12	7	17
Tamil	1	1	0	4
Muslims	1	1	0	0
Age at study entry				
30–60 years	9	7	2	16
> 60 years	12	7	5	5
Body Mass index				
Under weight	2	2	0	1
Ideal Weight	11	5	5	8
Overweight	6	4	2	7
Pre obese	2	2	0	3
Obese	0	0	0	2
Histological status of cancer				
Well differentiated adenocarcinoma	3	2	1	N/A
Moderately differentiated adenocarcinoma	10	6	4	N/A
Poorly differentiated adenocarcinoma	1	1	0	N/A
Unknown	7	5	2	N/A
Smoking history				
Yes	6	6	0	3
No	15	8	7	18
Alcohol consumption				
Yes	9	8	1	6
No	12	6	6	15
Betel-quid chewing				
Yes	10	8	2	3
No	11	6	5	18
Codon 72 polymorphism				
Arginine	6	5	1	6
Proline	6	3	3	5
Arginine/ Proline	9	6	3	10

### Analysis of TP53 sequence variants

A total of 16 sequence variants were found in 30 BC patients and 15 sequence variants were found in 21 CRC patients. In healthy male and female controls 6 and 8 variants were found respectively. Table 3 illustrates the characteristics of each variant and detailed *in-silico* and functional analysis are given in Additional file 2: Table S2.

**Table 3:** Prediction of pathogenicity of identified variants

No	HGVS Nomenclature		Location	Mutation type	No. of carriers in the study cohort				Novel or reported and the code reported in databases for reported variants	*Pathogenicity prediction: *In-silico* or functional	Conclusion
	cDNA	Protein			BC patients (*N* = 30)	CRC patients (*N* = 21)	Healthy controls – Males (*N* = 11)	Healthy controls - Females (*N* = 30)			
1	c.848_849delGC	p.Arg283Hisfs*22	E8	F	1	0	0	0	Novel	*In-silico*	Path
2	c.851_855delCAGAG	p.Thr284Argfs*20	E8	F	1	0	0	0	Novel	*In-silico*	Path
3	c.431_433delAGC	p.Gln144del	E5	IF	0	1	0	0	Novel	*In-silico*	Path
4	c.637C > T	p.Arg213*	E 6	NS	1	1	0	0	rs397516436	*In-silico*	Path
5	c.400 T > G	p.Phe134Val	E5	M	1	0	0	0	COSM43941	Both	Path
6	c.524G > A	p.Arg175His	E 5	M	0	1	0	0	rs28934578, COSM10648	Both	Path
7	c.581 T > G	p.Leu194Arg	E6	M	0	1	0	0	rs1057519998, COSM44571	Both	Path
8	c.730G > T	p.Gly244Cys	E7	M	1	0	0	0	COSM11524	Both	Path
9	c.733G > A	p.Gly245Ser	E7	M	0	2	0	0	rs28934575, COSM6932	Both	Path
10	c.743G > A	p.Arg248Gln	E7	M	3	0	0	0	rs11540652/ COSM10662	Both	Path
11	c.840A > T	p.Arg280Ser	E8	M	1	0	0	0	COSM44171	Both	Path
12	c.844C > T	p.Arg282Trp	E 8	M	0	1	0	0	rs28934574, COSM10704	Both	Path
13	c.626G > A	p.Arg209Lys	E6	M	1	0	0	0	COSM45995	Both	LP
14	c.63C > T	p.Asp21Asp	E 2	S	2	0	0	1	rs1800369	–	LB
15	c.459C > T	p.Pro153Pro	E 5	S	0	1	0	1	rs72661116, COSM43964	*In-silico*	LB
16	c.903A > G	p.Pro301Pro	E8	S	1	0	0	0	rs72661120/ COSM44165	*In-silico*	LB
17	c.-140G > A	–	E 1	3’UTR	1	2	0	0	novel	–	LB
18	c.97-29C > A	–	I 3	I	1	2	0	3	rs17883323	*In-silico*	US
19	c.74 + 16G > C	–	I 2	I	0	1	0	0	Novel	–	LB
20	c.74 + 38C > G	–	I 2	I	20	14	6	24	rs1642785	–	LB
21	c.96 + 41_96 + 56delACCTGGAGGGCTGGGG	–	I 3	I	28	6	11	27	rs59758982	–	LB
22	c.97-52G > A	–	I3	I	0	0	0	1	rs540683791	–	LB
23	c.782 + 72C > T	–	I 7	I	11	9	7	9	rs12947788	–	LB
24	c.782 + 92 T > G	–	I 7	I	11	9	7	9	rs12951053	–	LB
25	c.75-42G > A	–	I 2	I	0	0	1	0	Novel	–	LB
26	c.782 + 79C > T	–	I 7	I	0	0	1	0	Novel	–	LB
27	c.673-36G > C	–	I 6	I	0	1	0	0	rs17880604	*In-silico*	B

*Path* Pathogenic, *LP* Likely pathogenic, *US* variant with uncertain significance, *LB* Likely Benign, *B* Benign, *FS* Frameshift, *IF* In-frame, *M* Missense, *NS* Nonsense, *S* Silent, *I* Intron, *E* Exon, *3’UTR* 3′ Untranslated Region

*Details of *in-silico* and functional prediction are given in supplementary Table 2

**Fig. 1:**
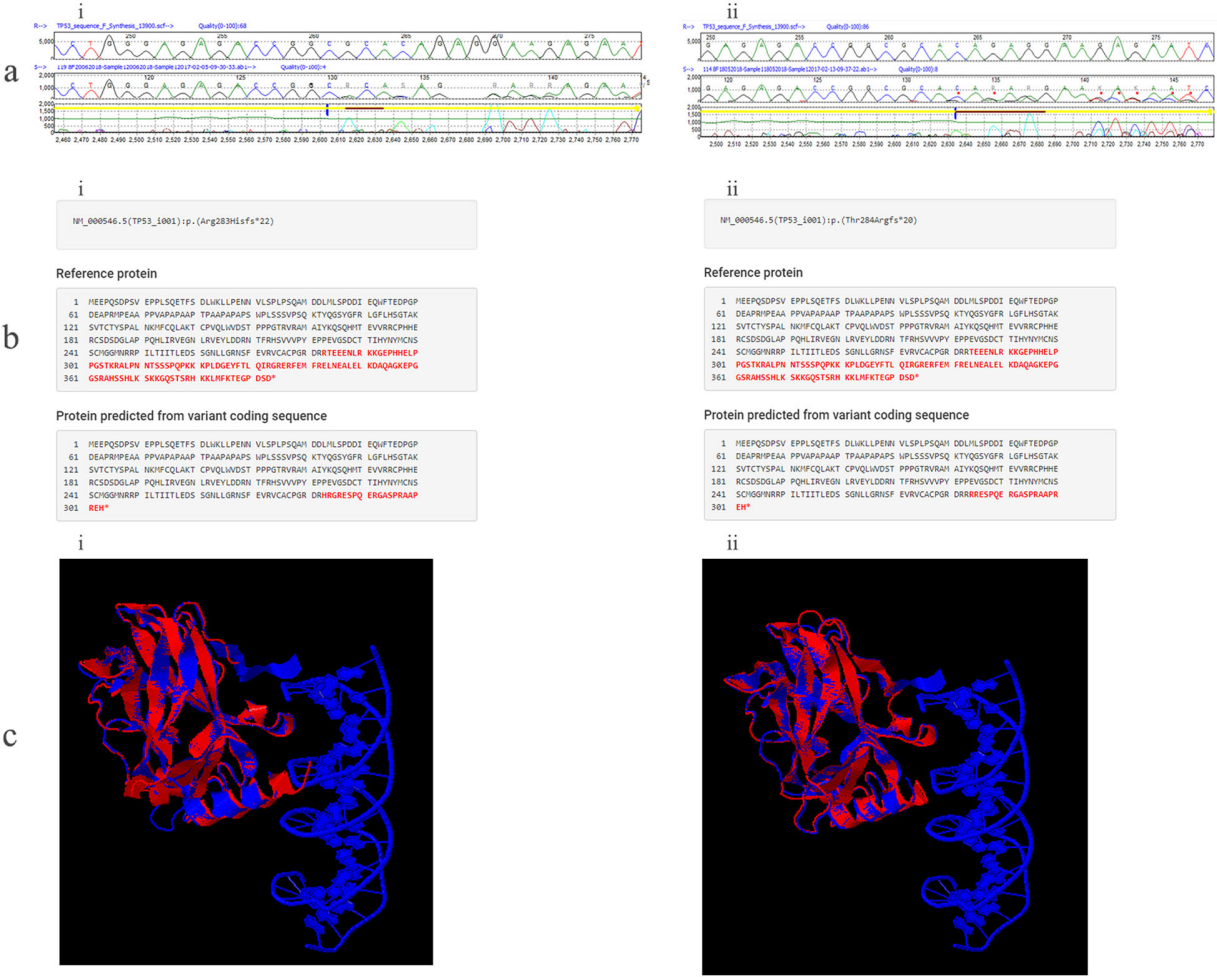
Novel frameshift deletions detected in exon 8. **a** Mutation Surveyor®V4.0.9 images indicating the heterozygous deletion point; R indicates the reference *TP53* sequence and S indicates the study sample *TP53* sequence. (i. - c.848_849delGC, ii - c.851_855delCAGAG). **b** Protein prediction using Mutalyzer 2.0.26. (i. - c.848_849delGC, ii - c.851_855delCAGAG). **c** Superimposed image of predicted mutated protein with wildtype p53 protein (PDB ID – 1TUP) Red indicates the mutated protein, Blue indicates the wildtype protein (i. - c.848_849delGC, ii - c.851_855delCAGAG)

Two novel frameshift variants were found in exon 8. The first of these, c.848_849delGC (Fig. 1), was observed in a 55 year old female BC patient with triple negative, poorly differentiated invasive ductal carcinoma, which results in an Arginine to Histidine substitution at the site of the deletion followed by other downstream amino acid changes and truncation of the protein at codon 303. The second frameshift variant, c.851_855delCAGAG (Fig. 1), was found in a 62 year old female BC patient with human epidermal growth factor receptor-2 (HER2) negative invasive ductal carcinoma with Ki67 index 32% and it result in a coding change from Threonine to Arginine at the site of deletion and other downstream amino acid encoding changes, plus a premature stop codon at position 302. Both the predicted truncated proteins are similar in length, with tetramerization and negative regulatory domains lost in both proteins.

**Fig. 2:**
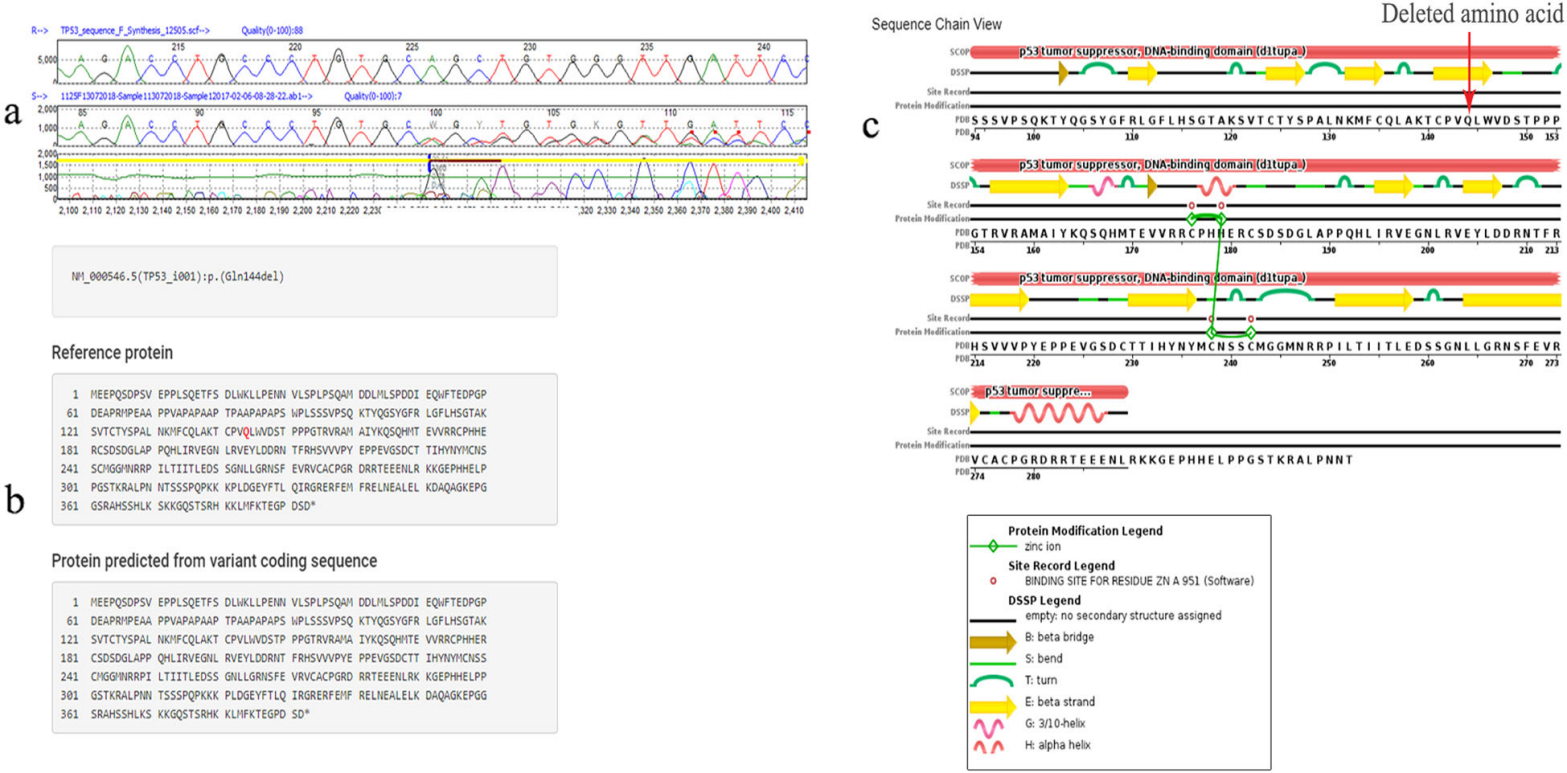
Novel in-frame deletion c.431_433delAGC detected in exon 5. **a** Mutation Surveyor®V4.0.9 images indicating the heterozygous deletion point; R indicates the reference *TP53* sequence and S indicates the study sample *TP53* sequence. **b** Protein prediction using Mutalyzer 2.0.26. **c** Sequence chain view of wild type p53 (PDB ID – 1TUP) protein showing the position of loss of the amino acid

A novel 3-base pair in-frame deletion was identified in exon 5. c.431_433delAGC (Fig. 2), resulting in the loss of the Glutamine amino acid residue at position 144 present in the β strand of the DNA binding domain and produces a 392 amino acid, shorter by one amino acid compared with the 393 amino acid full length protein. It was detected in a 66-year-old female CRC patient with moderately differentiated adenocarcinoma who had a previous history of cervix cancer. This patient also had a pathogenic *KRAS* codon 12 variant sequence (c.35G > A; rs121913529).

A reported nonsense variant, c.637C > T in exon 6 was found in both BC and CRC patients, resulting in a change from Arginine to a stop codon at 213. This truncated protein lacks part of the DNA binding domain, the tetramerization domain and negative regulatory domain, which would make the p53 protein non-functional. The BC patient was 47 years old with estrogen receptor (ER)/ progesterone receptor (PR) positive, HER2 equivocal invasive ductal carcinoma. The CRC patient was a 58 year old female with moderately differentiated adenocarcinoma.

There were four reported pathogenic missense variants, c.400 T > G, c.730G > T, c.743G > A, c.840A > T in exons 5, 7 and 8 observed only in BC patients. Variants c.400 T > G, c.730G > T and c.840A > T were observed in one patient each, while c.743G > A present in a CpG site was observed in 3 patients and 2 of them had triple negative invasive ductal carcinoma.

There were four reported pathogenic missense variants, c.524G > A, c.581 T > G, c.733G > A, c.844C > T in exon 5, 6, 7 and 8 respectively observed only in patients with CRC. A c.524G > A variant was observed in a 67 year old male patient with moderately differentiated adenocarcinoma. He also had the pathogenic *KRAS* codon 61 variant (c.183A > T; rs17851045). A c.581 T > G substitution was found in a 65 year old female with well differentiated adenocarcinoma. The missense variant c.733G > A was found in a 62 year old male patient with moderately differentiated adenocarcinoma and in a 71 year old female with tubular adenocarcinoma. A c.844C > T substitution was reported in a 66 year old female with moderately differentiated adenocarcinoma, who had a previous history of ovarian cancer. She also carried the pathogenic *KRAS* codon 12 variant (c.34G > C; rs121913530).

A likely pathogenic variant c.626G > A in exon 6 was observed in a 48 year old BC patient with ductal carcinoma. There were also 2 silent variants with uncertain significance, of which, c.63C > T appeared in 2 BC patients and in 1 female healthy control and c.459C > T appeared in 1 CRC patient and in a healthy control. Another silent variant c.903A > G observed in exon 8 in 1 BC patient is categorized as likely benign.

The codon 72 variant (p.R72P) in exon 4 is a well known *TP53* polymorphism. In the present study, R/R, R/P and P/P genotype distribution was 12 (40%), 11 (36.67%), 7 (23.33%) respectively in BC patients and 12 (40%), 13 (43.33%) and 5 (16.66%) respectively in healthy controls. No significant difference was observed (*p* = 0.78) in the prevalence of different genotypes in either the BC patients or the healthy controls. The genotypic distribution of R/R, R/P and P/P was 6 (28.57%), 9 (42.86%), 6 (28.57%) respectively in CRC patients and 6 (28.57%), 10 (47.62%) and 5 (23.81%) respectively in healthy controls. Similarly, there was also no significant difference (*p* value = 0.93) observed in the prevalence of different genotypes between the CRC patients and healthy controls.

### Immunohistochemistry

**Fig. 3:**
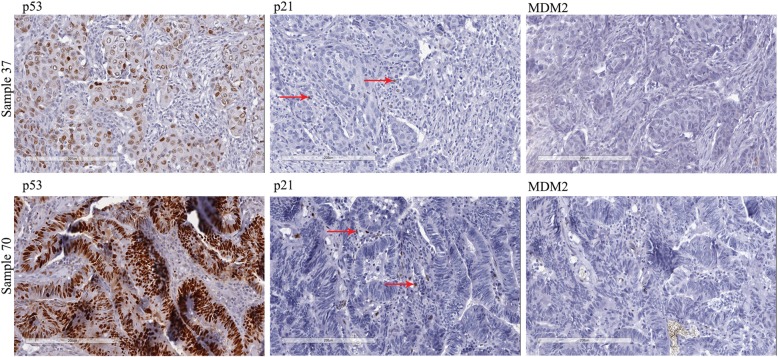
Different pattern of nuclear immunohistochemical expression of p53, p21 and MDM2 in **a** breast cancer tissue (Sample 37) and **b** colorectal cancer tissue (Sample 70) (Magnification 20X). p53 shows pattern A, p21 shows pattern B (red arrows indicate the rare positive cells) and MDM2 shows pattern **c** staining

The results obtained from immunohistochemistry analysis of 13 BC and 14 CRC tissue samples for IHC, were categorized into three; widespread IHC positive tumour nuclear staining involving either the entire or a segment of a tissue section (Pattern-A), rare/ scattered positive cells (Pattern-B) and complete absence of IHC positive signal (Pattern-C) (Fig. 3).

### Status of TP53 gene and the p53 protein expression

For BC patients, positive IHC staining was detected in 7/13 (53.85%) cases. Three of these tumour sections showed pattern A, while 4 showed Pattern-B. All 3 BC cases that showed Pattern-A had *TP53* missense variants while the 4 cases that showed Pattern-B had no detectable pathogenic variants of *TP53*. Among the 6 cases with immuno-negativity (Pattern-C), one had a silent variant and the remaining 5 patients had wild-type *TP53.*

For CRC patients, positive IHC staining was observed in 11/14 (78.57%) cases. Four of these tumours showed pattern A, while 7 showed pattern-B. Among the 4 CRC samples that showed pattern A, 3 had a *TP53* missense variant each, while the remaining case had no detectable pathogenic variants. All 7 cases that showed pattern-B had no detectable pathogenic *TP53* variants. Among the 3 cases with pattern-C, one had a non-sense variant, another one had a silent variant and the remaining patient showing immuno-negativity had wild-type *TP53*.

### Comparison of p53 protein expression with the expression p21 and MDM2

Among the 3 BC samples showing p53 IHC staining with pattern A; 1 showed pattern B staining and the remaining 2 showed pattern C for p21. From the 4 samples with pattern B of p53 expression, 3 showed pattern B, while 1 showed pattern C for p21. All 6 samples with pattern C IHC for p53 also showed pattern C for p21. However, expression of MDM2 for all samples showed pattern C IHC staining, regardless of the expression pattern of p53 or p21 protein (Table 4–1).

**Table 4:**
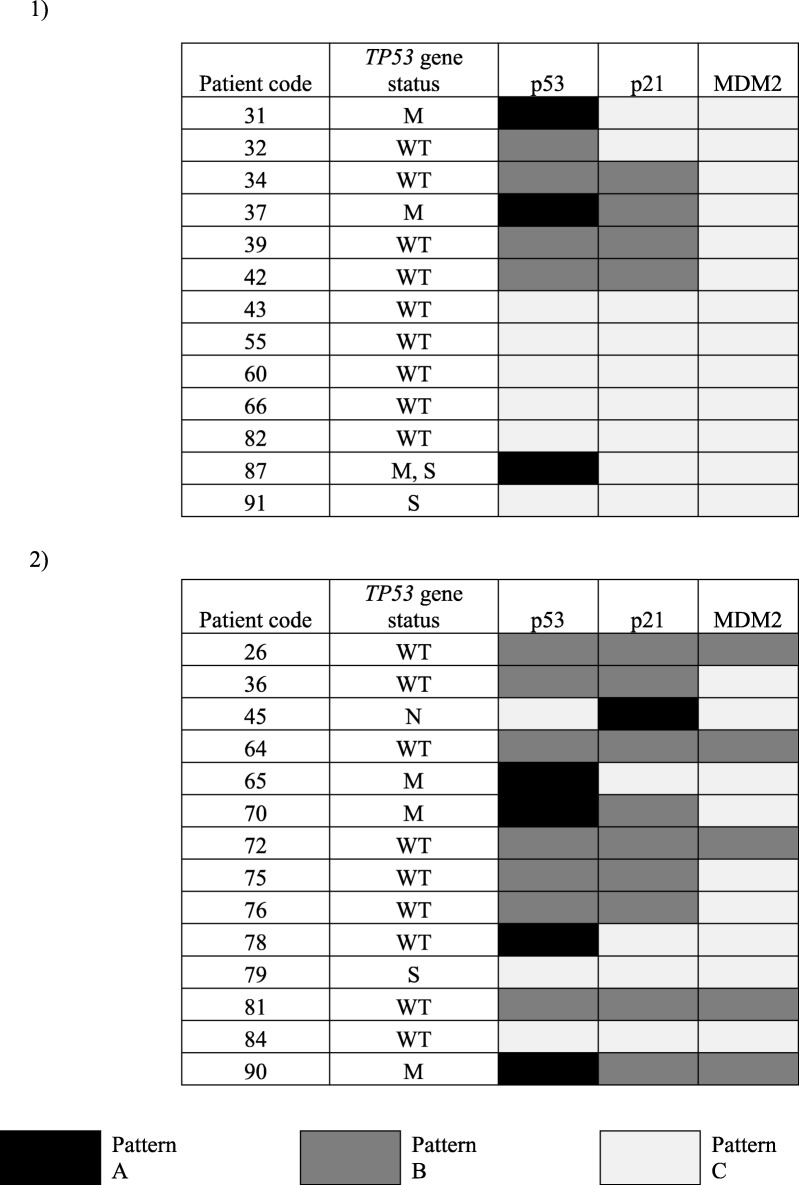
Correlation between p53, p21, MDM2 expression with *TP53* gene status in (1) breast cancer and (2) colorectal cancer.

Among 4 CRC samples with pattern A p53 IHC staining, 2 showed pattern B and the remaining 2 showed pattern C staining for p21. All 7 samples with pattern B p53 IHC staining showed pattern B staining for p21. Among 3 with pattern C p53 IHC staining, 2 showed pattern C staining for p21 and the remaining one showed pattern A for p21 IHC staining. In the case of MDM2, among the 4 samples with p53 IHC staining with pattern A, 1 showed pattern B and the remaining 3 showed pattern C. From the 7 samples with pattern B of p53 expression, 4 showed pattern B, while 3 showed pattern C for MDM2. All 3 samples with pattern C IHC for p53 also showed pattern C for MDM2 (Table 4–2).

## Discussion

This is a preliminary study focused on the mutation spectrum of the *TP53* gene and expression of p53 and downstream p53 transcriptional proteins for a series of Sri Lankan BC and CRC patients.

Invasive ductal and lobular BCs are the major malignancies of the breast and account for approximately 80 and 15% of cases respectively [17]. In our study cohort, 96.5% were ductal carcinoma and only 3.5% were the lobular carcinoma. The frequency of pathogenic *TP53* variation in the BC patient cohort (31%) was closer to figures reported in the cBioportal and IARC TP53 databases (32 and 22.8% respectively). But the frequency of pathogenic TP53 variation in the colorectal cancer cohort (33.3%) was lower compared to what has been reported in the cBioportal (53.4%) and IARC TP53 database (43.3%) [15, 18, 19].

The incidence of overweight and obesity is increasing significantly worldwide, and it is associated with obesity-related cancers, including postmenopausal BC and CRC [20]. In our study cohort 63.3% of the BC patients and 38.1% of the CRC patients were either overweight, pre-obese or obese based on Asian criteria for BMI cut-off.

### Variant analysis

All the 13 variants altering proteins are found between exons 5 to 8 and 9 (69%) of them are missense variants. It is supported by the data provided in IARC TP53 database as approximately 95% of the pathogenic variants are found between exons 5 to 8 and 73% of the alterations are missense variants. According to cosmic database, 53.14 and 52.83% of the alterations found in breast and colorectal cancers respectively are missense variants [6, 15].

Among the pathogenic variants found, the frameshift variants; c.848_849delGC, c.851_855delCAGAG and the in-frame variant; c.431_433delAGC have not been previously reported. The c.637C > T, nonsense variant has been commonly reported in colon, breast, oesophageous, skin, and stomach cancers. The somatic missense variants; c.400 T > G, c.524G > A, c.581 T > G, c.730G > T, c.733G > A, c.743G > A, c.840A > T and c.844C > T have been reported in various cancers previously. However, c.730G > T and c.840A > T found in our BC patient cohort have been not previously reported in any type of BCs. On the other hand, c.524G > A, c.733G > A and 844C > T found in the CRC patient cohort have been previously reported with a high prevalence in CRC cases. The likely pathogenic variant, c.626G > A, observed in our BC cohort also has been reported in various cancers but not in BC. The c.63C > T silent variant is reported only in the IARC TP53 database and the details are not provided. The c.459C > T variant has been observed previously in BC but not in CRC, whereas c.903A > G has been reported only in oesophageal and central nervous system cancers [6, 15].

The exonic variants c.637C > T, c.524G > A, c.844C > T, c.63C > T, c.459C > T and c.-140G > A found in this study were also observed in head and neck cancer patients by us [14]. Comparison of *TP53* variants found in BCs with those in CRCs in this study showed c.637C > T and c.-140G > A were the only two exonic variants found in common. Among the seven pathogenic *TP53* variants found in CRC patients in the present cohort, only two were present in males suggesting a higher prevalence of pathogenic *TP53* variation in females when compared to males in CRC, although larger number of patient samples would need to be analysed to confirm this.

The most important factors that control the regulation of normal breast cell growth are p53 and hormone receptors PR, ER and HER-2. In normal breast tissues, cell proliferation is promoted by oestrogen. Stimulation of oestrogen also increases the level of p53 by both increasing the transcription and stabilization of p53. The increased level of p53 counters enhanced cell proliferation, thus keeping the proliferation and cell death in balance. However, in the cells where the p53 and ER levels are deregulated, the equilibrium between the cell proliferation and cell death is disturbed which leads to the uncontrolled tumour growth. Similarly, even PR and HER2 have functional interactions with mutant p53 [1, 21, 22]. Studies have reported that of the tumours with *TP53* mutations, 55% are ER and PR negative, and 57% to be HER2 negative [23]. According to the Multi-Omics Breast Cancer Database, all 3 patients reported with a tumour somatic c.743G > A variant had a triple negative hormone status where the BC patient lacks ER, PR expression as well as HER2 expression [24]. In our study, 2 out of the 3 patients detected with a c.743G > A variant were triple negative and the other patient’s receptor status is unknown. In addition, a patient with a c.848_849delGC variant tumour also had triple negative BC diagnosed.

Analysis of *KRAS*, *BRAF* and *PI3KCA* genes helps in the prognosis as well as in the treatment of colorectal cancer [25]. Out of seventeen CRC patients analysed for *KRAS*, *BRAF* and *PIK3CA*, none of them had *BRAF* or *PIK3CA* pathogenic hotspot variant sequences, but 4 had pathogenic variants in *KRAS*; three in codon 12 and one in codon 61. Out of these 4 patients, three had pathogenic *TP53* variants; one had a previous history of cervical cancer and another had a previous history of ovarian cancer.

There are controversial opinions on the association of cancer risk and prognosis with codon 72 polymorphism, as some studies suggests that codon 72 Arginine has a protective effect based on a greater apoptotic potential [26–28] while others failed to replicate these findings [29, 30]. In the present study neither allele of p.R72P was significantly associated with BC or CRC. Similarly in our previous studies no significant association was found between the codon 72 polymorphism and head and neck cancer [14]. However, the number of patients in the current study is too small to draw definitive negative conclusions about any association with cancer risk and prognosis.

### Immunohistochemical analysis

Wild type p53 has a very short half – life, as it is generally kept under tight auto-regulatory control by MDM2. But in the case of mutant p53, the p53-dependent MDM2 expression is lost and mutant forms of p53 are no longer recognised by MDM2. The breakdown of the MDM2 mediated negative feedback loop leads to the accumulation of mutant p53 [31, 32]. All 6 missense variants identified in the current study were associated with the accumulation of mutant p53 protein resulting in the Pattern-A IHC staining, whereas all the samples (*N* = 11) with Pattern-B IHC were *TP53* wild-type. Six other wild-type samples, two samples with silent mutations and one sample with a nonsense variant showed no positive immuno-reactivity. Similar results were observed in our previous study on head and neck cancer [14]. *TP53* missense mutations are strongly associated with the strong immuno positivity of p53 protein in breast (*p*-value = 0.001417) and breast and colorectal cancer combined (p-value = 8.104e-06). Several previous studies which observed an association between *TP53* mutations and higher expression of p53 protein in various types of cancer [33, 34] also support our findings. A larger study done on 7878 variants representing 60 distinct tumour sites from the IARC TP53 Database concludes that missense mutations are IHC positive while nonsense mutations, frameshift mutations and deletions were immunonegative [35].

A number of studies have examined the relationship between the expression pattern of p53 and p21 in various human tumours with conflicting results. Some studies showed that abnormal p53 (overexpression) was associated with low or complete absence of expression of p21 [36–38], while others showed no significant correlation between p21 expression and the abnormal accumulation of p53 [39, 40]. Lack of correlation of p53 and p21 may be either due activation of p21 via p53 independent pathways or due to some p53 mutants being still able to transcriptionally turn on the p21 protein [37]. Another important transcriptional target of p53 is MDM2. However, there are limited number of studies focused on the association of expression between p53 and MDM2. According to those studies, higher expression of both p53 and MDM2 was observed in most of the pancreatic cancer and sarcoma cases examined [41, 42]. This overexpression of MDM2 may be due to p53-independent transcriptional activation even in the presence of abnormal p53. In addition, functional studies have reported another novel mechanism of MDM2 stabilization and accumulation. MDM2 in tumour cells may be stabilized by interaction with mutant p53 and thus lead to accumulation [43]. We attempted to investigate the functional activity of p53 by analysing the expression of the downstream proteins of p53 namely, p21 and MDM2 in our study cohort. There is no significant correlation observed between *TP53* gene status and p21 protein staining in breast cancer (*p*-value = 0.9214) and in colorectal cancer (p-value = 0.6426). Similarly, no significant correlation was found between *TP53* gene status and MDM2 protein both in breast cancer (p-value = 0.0522) and colorectal cancer (p-value = 0.6914). According to Spearman’s Rank Correlation, there are no significant correlations in the expression of p21 and MDM2 in relation to expression of p53. This non-consistent pattern in the expression of p21 and MDM2 in relation to expression of p53 and mutational status of *TP53* may be due to the p53 independent pathways of p21 and MDM2 activation and the limited sample size. Thus, analysis of p21 and MDM2 protein expression in combination with p53 protein expression had no added advantage in differentiating between normal and mutant p53 protein.

## Conclusion

We examined all exons and splicing sites of the *TP53* gene in BC and CRC in a cohort of Sri Lankan patients and found a high occurrence of gene alterations including several novel variants. All p53 protein altering variants found were positioned between exons 5–8. Only the point missense variants were associated with strong immuno-positive staining for p53. *TP53* wild type samples were associated with either rare isolated positively staining cells on tumour sections or complete absence of positive signal. Both truncating and silent variants were associated with the absence of positive IHC staining for p53. However there was no significant correlation found between the expression of p21 and MDM2 with the expression of p53.

## Supplementary information

**Additional file 1: Table S1.** Optimised concentration, incubation time of the primary antibodies and antigen retrieval buffer

**Additional file 2: Table S2.** *In-silico* and functional prediction of identified variants

## Data Availability

The datasets used and/or analysed during the current study are available from the corresponding author on reasonable request.

## Abbreviations

BC: Breast cancer
CRC: Colorectal cancer
ER: Estrogen Receptor
FFPE: Formalin Fixed Paraffin Embedded
HER2: Human Epidermal growth factor Receptor-2
IHC: Immunoistochemistry
PCR: Polymerase Chain Reaction
PR: Progesterone Receptors

## References

[1] Walerych D., Napoli M., Collavin L., Del Sal G. (2012). The rebel angel: mutant p53 as the driving oncogene in breast cancer. Carcinogenesis.

[2] Arnold Melina, Sierra Mónica S, Laversanne Mathieu, Soerjomataram Isabelle, Jemal Ahmedin, Bray Freddie (2016). Global patterns and trends in colorectal cancer incidence and mortality. Gut.

[3] Cancer Incidence Data, Sri Lanka 2010. In Edited by Programme NCC Sri Lanka.

[4] BREAST CANCER.ORG. https://www.breastcancer.org/

[5] Haggar Fatima, Boushey Robin (2009). Colorectal Cancer Epidemiology: Incidence, Mortality, Survival, and Risk Factors. Clinics in Colon and Rectal Surgery.

[6] Tate John G, Bamford Sally, Jubb Harry C, Sondka Zbyslaw, Beare David M, Bindal Nidhi, Boutselakis Harry, Cole Charlotte G, Creatore Celestino, Dawson Elisabeth, Fish Peter, Harsha Bhavana, Hathaway Charlie, Jupe Steve C, Kok Chai Yin, Noble Kate, Ponting Laura, Ramshaw Christopher C, Rye Claire E, Speedy Helen E, Stefancsik Ray, Thompson Sam L, Wang Shicai, Ward Sari, Campbell Peter J, Forbes Simon A (2018). COSMIC: the Catalogue Of Somatic Mutations In Cancer. Nucleic Acids Research.

[7] The TP53 website. 2017. https://p53.fr/.

[8] Kandioler-Eckersberger D, Ludwig C, Rudas M, Kappel S, Janschek E, Wenzel C (2000). TP53 mutation and p53 overexpression for prediction of response to neoadjuvant treatment in breast cancer patients. Clin Cancer Res.

[9] Olivier Magali, Hainaut Pierre (2001). TP53 mutation patterns in breast cancers: searching for clues of environmental carcinogenesis. Seminars in Cancer Biology.

[10] Hill Kathleen A., Sommer Steve S. (2002). p53 As a mutagen test in breast cancer. Environmental and Molecular Mutagenesis.

[11] Lu Yunxia, Segelman Josefin, Nordgren Ann, Lindström Lina, Frisell Jan, Martling Anna (2016). Increased risk of colorectal cancer in patients diagnosed with breast cancer in women. Cancer Epidemiology.

[12] Andersson Michael, Jensen Maj-Britt, Engholm Gerda, Henrik Storm Hans (2008). Risk of second primary cancer among patients with early operable breast cancer registered or randomised in Danish Breast Cancer cooperative Group (DBCG) protocols of the 77, 82 and 89 programmes during 1977–2001. Acta Oncologica.

[13] Tang L Y L, Nugent Z, Demers A A, Singh Harminder (2009). Incidence of Right-Sided Colorectal Cancer After Breast Cancer: A Population-Based Study. The American Journal of Gastroenterology.

[14] Manoharan V, Karunanayake EH, Tennekoon KH, De Silva S, De Silva K, Angunawela P, Lunec J. Nucleotide variants and protein expression of TP53 in a Sri Lankan cohort of patients with head and neck cancer. Mol Med Report 2019;19:2781–2791. https://doi.org/10.3892/mmr.2019.9948. 10.3892/mmr.2019.9948 PMC6423636 30816478

[15] Bouaoun Liacine, Sonkin Dmitriy, Ardin Maude, Hollstein Monica, Byrnes Graham, Zavadil Jiri, Olivier Magali (2016). TP53Variations in Human Cancers: New Lessons from the IARC TP53 Database and Genomics Data. Human Mutation.

[16] Richards Sue, Aziz Nazneen, Bale Sherri, Bick David, Das Soma, Gastier-Foster Julie, Grody Wayne W., Hegde Madhuri, Lyon Elaine, Spector Elaine, Voelkerding Karl, Rehm Heidi L. (2015). Standards and guidelines for the interpretation of sequence variants: a joint consensus recommendation of the American College of Medical Genetics and Genomics and the Association for Molecular Pathology. Genetics in Medicine.

[17] Sainsbury J. R. C. (1996). Diseases of the breast. J. R. Harris, M. E. Lippman, M. Morrow and S. Hellman (eds). 285 × 225 mm. Pp. 1047. Illustrated. 1996. Philadelphia, Pennsylvania: Lippincott-Raven. £118. British Journal of Surgery.

[18] Cerami Ethan, Gao Jianjiong, Dogrusoz Ugur, Gross Benjamin E., Sumer Selcuk Onur, Aksoy Bülent Arman, Jacobsen Anders, Byrne Caitlin J., Heuer Michael L., Larsson Erik, Antipin Yevgeniy, Reva Boris, Goldberg Arthur P., Sander Chris, Schultz Nikolaus (2012). The cBio Cancer Genomics Portal: An Open Platform for Exploring Multidimensional Cancer Genomics Data: Figure 1. Cancer Discovery.

[19] Gao J., Aksoy B. A., Dogrusoz U., Dresdner G., Gross B., Sumer S. O., Sun Y., Jacobsen A., Sinha R., Larsson E., Cerami E., Sander C., Schultz N. (2013). Integrative Analysis of Complex Cancer Genomics and Clinical Profiles Using the cBioPortal. Science Signaling.

[20] Berger Nathan A. (2014). Obesity and cancer pathogenesis. Annals of the New York Academy of Sciences.

[21] Berger C., Qian Y., Chen X. (2013). The p53-Estrogen Receptor Loop in Cancer. Current Molecular Medicine.

[22] Casalini Patrizia, Botta Lorena, Ménard Sylvie (2001). Role of p53 in HER2-induced Proliferation or Apoptosis. Journal of Biological Chemistry.

[23] Pereira B, Chin S-F, Rueda OM, Vollan H-KM, Provenzano E, Bardwell HA, et al. The somatic mutation profiles of 2,433 breast cancers refine their genomic and transcriptomic landscapes. Nat Commun 2016;7. https://doi.org/10.1038/ncomms11479. 10.1038/ncomms11479 PMC4866047 27161491

[24] Xie Bingbing, Yuan Zifeng, Yang Yadong, Sun Zhidan, Zhou Shuigeng, Fang Xiangdong (2018). MOBCdb: a comprehensive database integrating multi-omics data on breast cancer for precision medicine. Breast Cancer Research and Treatment.

[25] Zhao B, Wang L, Qiu H, Zhang M, Sun L, Peng P, et al. Mechanisms of resistance to anti-EGFR therapy in colorectal cancer. Oncotarget. 2017;8:3. https://doi.org/10.18632/oncotarget.14012. 10.18632/oncotarget.14012 PMC5354808 28002810

[26] Dumont Patrick, Leu J. I-Ju, Della Pietra Anthony C., George Donna L., Murphy Maureen (2003). The codon 72 polymorphic variants of p53 have markedly different apoptotic potential. Nature Genetics.

[27] Toyama T, Zhang Z, Nishio M, Hamaguchi M, Kondo N, Iwase H, et al. Association of TP53 codon 72 polymorphism and the outcome of adjuvant therapy in breast cancer patients. Breast Cancer Res 2007;9:3. https://doi.org/10.1186/bcr1682. 10.1186/bcr1682 PMC1929098 17537232

[28] Damin Andrea P.S., Frazzon Ana P.G., Damin Daniel C., Roehe Adriana, Hermes Vanessa, Zettler Claudio, Alexandre Claudio O.P. (2006). Evidence for an association of TP53 codon 72 polymorphism with breast cancer risk. Cancer Detection and Prevention.

[29] Baynes C, Healey CS, Pooley KA, Scollen S, Luben RN, Thompson DJ, et al. Common variants in the ATM, BRCA1, BRCA2, CHEK2 and TP53 cancer susceptibility genes are unlikely to increase breast cancer risk. Breast Cancer Res 2007;9:2. https://doi.org/10.1186/bcr1669. 10.1186/bcr1669 PMC1868915 17428325

[30] MABROUK IMED, BACCOUCHE SAMI, EL-ABED RYM, MOKDAD-GARGOURI RAJA, MOSBAH ALI, SAÏD SALEM, DAOUD JAMEL, FRIKHA MOUNIR, JLIDI RACHID, GARGOURI ALI (2003). No Evidence of Correlation between p53 Codon 72 Polymorphism and Risk of Bladder or Breast Carcinoma in Tunisian Patients. Annals of the New York Academy of Sciences.

[31] Reich N C, Oren M, Levine A J (1983). Two distinct mechanisms regulate the levels of a cellular tumor antigen, p53. Molecular and Cellular Biology.

[32] Blagosklonny Mikhail V (1997). Loss of function and p53 protein stabilization. Oncogene.

[33] Liu Jiangbo, Li Wei, Deng Miao, Liu Dechun, Ma Qingyong, Feng Xiaoshan (2016). Immunohistochemical Determination of p53 Protein Overexpression for Predicting p53 Gene Mutations in Hepatocellular Carcinoma: A Meta-Analysis. PLOS ONE.

[34] Liu Zhixian, Jiang Zehang, Gao Yingsheng, Wang Lirui, Chen Cai, Wang Xiaosheng (2019). TP53 Mutations Promote Immunogenic Activity in Breast Cancer. Journal of Oncology.

[35] Murnyák B, Hortobágyi T. Immunohistochemical correlates of TP53 somatic mutations in cancer. Oncotarget. 2016;7(40):64910. https://doi.org/10.18632/oncotarget.11912. 10.18632/oncotarget.11912 PMC5323125 27626311

[36] DOGLIONI CLAUDIO, PELOSIO PAOLA, LAURINO LICIA, MACRI ETTORE, MEGGIOLARO ENZO, FAVRETTI FRANCO, BARBARESCHI MATTIA (1996). p21/WAF1/CIP1 EXPRESSION IN NORMAL MUCOSA AND IN ADENOMAS AND ADENOCARCINOMAS OF THE COLON: ITS RELATIONSHIP WITH DIFFERENTIATION. The Journal of Pathology.

[37] Bukholm I. K., Nesland J. M., Kâresen R., Jacobsen U., Børresen A. L. (1997). Relationship between abnormal p53 protein and failure to express p21 protein in human breast carcinomas. The Journal of Pathology.

[38] Thor Ann D., Liu Shuquing, Moore II Dan H., Shi Qiuju, Edgerton Susan M. (2000). p21WAF1/CIP1 Expression in breast cancers: associations with p53 and outcome. Breast Cancer Research and Treatment.

[39] Yasd Wataru, Akama Yoshihiko, Yokozaki Hiroshi, Semba Shuho, Kudo Yasusei, Shimamoto Fumio, Tahara Eiichi (1997). Expression of p21WAF1/CIP1in colorectal adenomas and adenocarcinomas and its correlation with p53 protein expression. Pathology International.

[40] Marchetti A, Doglioni C, Barbareschi M, Buttitta F, Pellegrini S, Bertacca G (1996). p21 RNA and protein expression in non-small cell lung carcinomas: evidence of p53-independent expression and association with tumoral differentiation. Oncogene.

[41] Dong Ming (2005). Clinicopathological significance of p53 and mdm2 protein expression in human pancreatic cancer. World Journal of Gastroenterology.

[42] Ozgur T, Ugras N, Yalcinkaya U, Arici A (2013). Immunohistochemical detection of p53 and MDM2 expressions in liposarcoma with World health organization classification. Indian Journal of Cancer.

[43] Peng Yanhua, Chen Lihong, Li Changgong, Lu Wenge, Agrawal Sudhir, Chen Jiandong (2001). Stabilization of the MDM2 Oncoprotein by Mutant p53. Journal of Biological Chemistry.

