# Supplementary material for: Pattern of nucleotide variants of TP53 and their correlation with the expression of p53 and its downstream proteins in a Sri Lankan cohort of breast and colorectal cancer patients
Source: BMC Cancer. 2020 Jan 30;20:72. doi: 10.1186/s12885-020-6573-5 (PMC6990524; doi:10.1186/s12885-020-6573-5)
Supplement: Supplementary file 1 — Additional file 1: Table S1. Optimised concentration, incubation time of the primary antibodies and antigen retrieval buffer [file 12885_2020_6573_MOESM1_ESM.doc]

Additional file 1

Table S1. Optimised concentration, incubation time of the primary antibodies and antigen retrieval buffer

| Protein | Antigen retrieval buffer | Primary antibody | Concentration | Incubation time |
| --- | --- | --- | --- | --- |
| P53 | Citrate, PH = 6 | DO-7, DaKo | 1:100 | 1 hour |
| P21 | Citrate, PH = 6 | 2947S, Cell Signaling | 1:50 | 1 hour |
| MDM2 | Citrate, PH = 6 | OP46, MerkMillipore | 1:50 | 1 hour |
