# Supplementary material for: Pattern of nucleotide variants of TP53 and their correlation with the expression of p53 and its downstream proteins in a Sri Lankan cohort of breast and colorectal cancer patients
Source: BMC Cancer. 2020 Jan 30;20:72. doi: 10.1186/s12885-020-6573-5 (PMC6990524; doi:10.1186/s12885-020-6573-5)
Supplement: Supplementary file 2 — Additional file 2: Table S2. In-silico and functional prediction of identified variants [file 12885_2020_6573_MOESM2_ESM.doc]

Additional file 2

Table S2. *In-silico* and functional prediction of identified variants

| No | HGVS Nomenclature | *In - silico* prediction | | | | | | | NCBI | Prediction of functional activity | | | Conclusion |
| --- | --- | --- | --- | --- | --- | --- | --- | --- | --- | --- | --- | --- | --- |
|  | cDNA | Splice site | CpG site | Align GVGD | Mutation taster | Provean | SIFT | PolyPhen 2 |  | Transcriptional activity [21] | Functional activity prediction [22] | Dominant Negative Effect |  |
| 1 | c.848_849delGC | -- | -- | -- | DC | -- | -- | -- | -- | -- | -- | -- | Path |
| 2 | c.851_855delCAGAG | -- | -- | -- | DC | -- | -- | -- | -- | -- | -- | -- | Path |
| 3 | c.431_433delAGC | - | - | - | DC | - | - | - | - | - | - | - | Path |
| 4 | c.637C>T | -- | yes | -- | -- | - | - | -- | Path | -- | -- | -- | Path |
| 5 | c.400T>G | No | No | C45 | DC | D | D | D | -- | NF | NF | -- | Path |
| 6 | c.524G>A | -- | yes | C25 | DC | D | D | PO | Path | NF | NF | DN | Path |
| 7 | c.581T>G | -- | -- | C65 | DC | D | D | PR | US | NF | NF | -- | Path |
| 8 | c.730G>T | No | No | C65 | DC | D | D | D | LP | NF | NF | -- | Path |
| 9 | c.733G>A | -- | yes | C55 | DC | D | D | PR | Path | NF | NF | DN | Path |
| 10 | c.743G>A | No | Yes | C35 | DC | D | D | D | Pat | NF | NF | Yes | Path |
| 11 | c.840A>T | No | No | C65 | DC | D | D | D | -- | NF | NF | Yes | Path |
| 12 | c.844C>T | -- | yes | C65 | DC | D | D | PR | Path | NF | NF | MD | Path |
| 13 | c.626G>A | No | No | C0 | P | N | T | B | -- | F | F | -- | LP |
| 14 | c.63C>T | -- | -- | -- | -- | - | - | -- | -- | -- | -- | -- | LB |
| 15 | c.459C>T | -- | -- | -- | -- | - | - | -- | LB | -- | -- | -- | LB |
| 16 | c.903A>G | No | No | -- | P | N | T | -- | LB | -- | -- | -- | LB |
| 17 | c.-140G>A | -- | -- | -- | -- | - | - | -- | -- | -- | -- | -- | LB |
| 18 | c.97-29C>A | Al | -- | -- | -- | - | - | -- | -- | -- | -- | -- | US |
| 19 | c.74+16G > C | -- | -- | -- | -- | - | - | -- | -- | -- | -- | -- | LB |
| 20 | c.74+38C>G | -- | -- | -- | -- | - | - | -- | -- | -- | -- | -- | LB |
| 21 | c.96+41_96+56delACCTGGAGGGCTGGGG | -- | -- | -- | -- | - | - | -- | -- | -- | -- | -- | LB |
| 22 | c.97-52G>A | -- | -- | -- | -- | -- | - | -- | -- | -- | -- | -- | LB |
| 23 | c.782+72C>T | -- | -- | -- | -- | - | - | -- | -- | -- | -- | -- | LB |
| 24 | c.782+92T>G | -- | -- | -- | -- | - | - | -- | -- | -- | -- | -- | LB |
| 25 | c.75-42G>A | -- | -- | -- | -- | - | - | -- | -- | -- | -- | -- | LB |
| 26 | c.782+79C>T | -- | -- | -- | -- | - | - | -- | -- | -- | -- | -- | LB |
| 27 | c.673-36G>C | -- | -- | -- | -- | - | - | -- | B | -- | -- | -- | B |

DC - Disease causing, P – Polymorphism, N – Neutral, T – Tolerated, PR - Probably damaging, PO - Possibly Damaging, Path – Pathogenic, LP – Likely pathogenic, US - variant with uncertain significance, LB - Likely Benign, B – Benign, F – Functional, PF – Partially functional, D – Deleterious, NF – Non-functional, Al – Alter the splice site, Dominant Negative Effect – DN, Moderate DNE – MD, non DNE – ND

Variants were classified as ‘non-functional’ if the median is <=20, ‘Partially functional’ if the median is >20 and <=75, ‘functional’ if the median is >75 and <=140, and ‘supertrans’ if the median is >140based on the median of 8 promoter – specific activities, expressed as percent of wild-type protein and were categorized as ‘‘DNE’’ if they were Dominant Negative on both *p21^WAF1^* and RGC promoters or on all other promoters, ‘‘moderate-DNE’’ if they were Dominant Negative on some promoters and not on others, and ‘‘non-DNE’’ if they were not Dominant Negative on both WAF1 and RGC promoters, or none of the promoters [18, 19]
